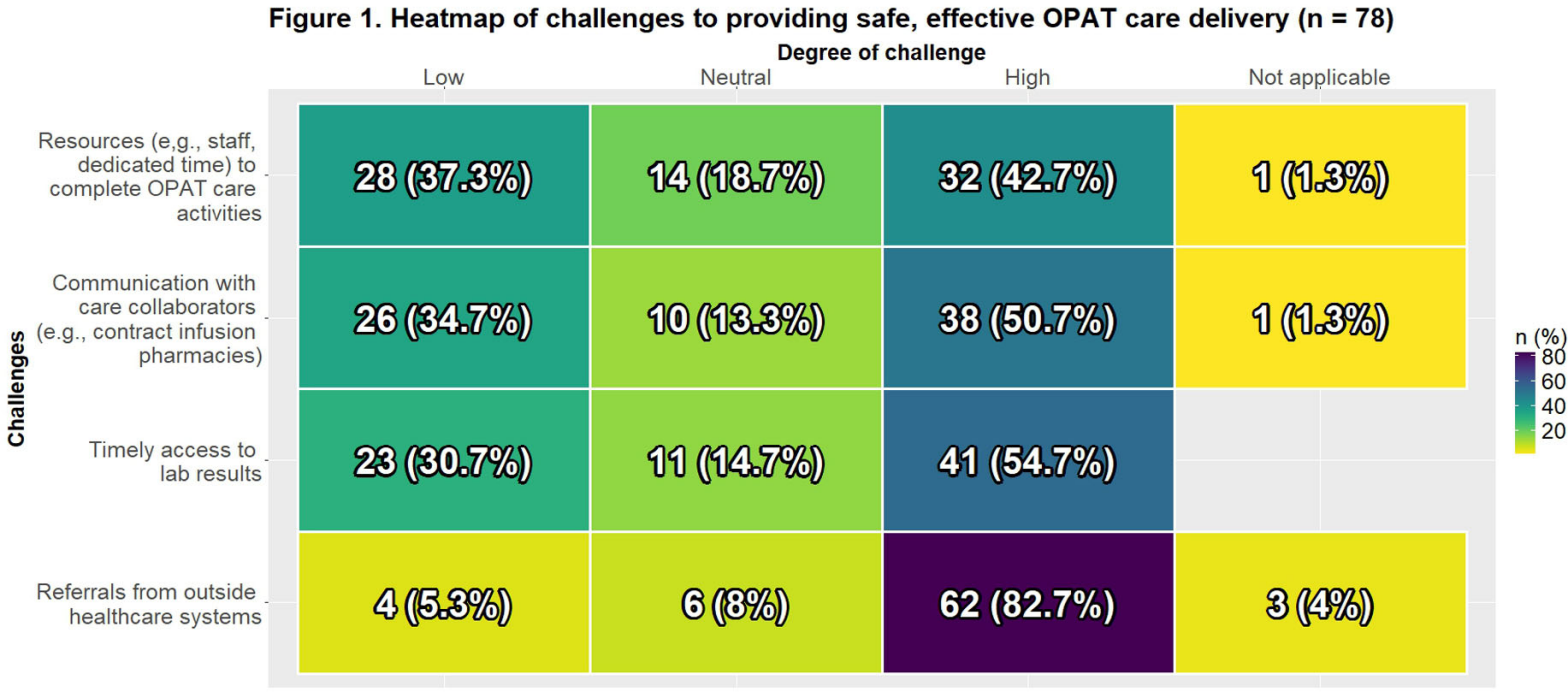# 341 Implementation of an Antimicrobial Stewardship Program in a Private Hospital: Clinical, Microbiological, and Economic Outcomes

**DOI:** 10.1017/ash.2026.10682

**Published:** 2026-06-23

**Authors:** Manon Nitta, Danielle Helminski, Elizabeth Scruggs-Wodkowski, Sara Keller, Ronald Kendall, Amanda Blok, Lauren Gauntlett, Nicholas Henry, Allison Ranusch, Milisa Manojlovich, Molly Harrod, Sarah Krein

**Affiliations:** 1 VA Ann Arbor Center for Clinical Management Research; 2 Center for Clinical Management Research, VA Ann Arbor Healthcare System; 3 VA Ann Arbor Healthcare System; 4 Johns Hopkins University; 5 VA Center for Clinical Management Research; 6 Ann Arbor VA Hospital Medical Center; 7 University of Michigan; 8 VA Ann Arbor Healthcare System, Center for Clinical Management Research; 9 VA Ann Arbor Hlthcare System and University of Michigan

## Abstract

**Background:** Outpatient parenteral antimicrobial therapy (OPAT) is a common approach to treating complex infections, offering benefits of higher patient satisfaction, reduced risk of hospital-acquired infections, and lower healthcare costs. However, limited information exists regarding how OPAT is delivered across U.S. healthcare systems. We sought to understand OPAT practices and identify facilitators and barriers to OPAT delivery at Veterans Affairs (VA) medical centers (VAMCs), which constitute the largest integrated healthcare system in the U.S. **Methods:** We conducted a national survey of VAMCs between January-April 2025. OPAT providers were invited via email to complete an online Qualtrics survey. Topics included care delivery, patient monitoring, and perceived challenges to safe, effective OPAT care. Data were summarized using descriptive statistics. **Results:** Of 139 surveys, 106 (76.3%) were completed and analyzed. Most VAMCs offered OPAT (78/106; 73.6%). Among those, 60/78 (76.9%) designated OPAT providers to monitor and manage care, often Infectious Disease (ID) physicians (54/60; 90.0%) and ID pharmacists (44/60; 73.3%). Dedicated time for OPAT care activities varied by site and role. Guidelines for OPAT care were reported at 45/78 (57.7%) VAMCs, typically outlining eligibility criteria as well as protocols for patient monitoring and follow-up. Generally, VAMCs required ID consultation prior to discharge and discharged 1-10 patients on OPAT per month, most of whom were followed by ID providers. OPAT-related outcomes were measured by about half of VAMCs (40/78; 52.6%); adverse events were most frequently measured. OPAT delivery extended beyond VAMCs through referrals to outside healthcare systems and collaboration with contract infusion pharmacies and home health agencies (Table 1). Respondents noted challenges to providing safe OPAT care, including dedicated time, communication with organizations outside the VA, and timeliness of labs (Figure 1). **Conclusions:** While individual VAMCs varied in delivery practices, key elements across programs included designated providers and defined OPAT care components. Challenges were driven by fragmented care, which hindered communication and coordination. Expanding VAMC capacity to provide OPAT and monitor patients may streamline communication, delivery, and continuity. A process map for referrals outside the VA may help designate roles and responsibilities for monitoring and providing OPAT care.

**Figure:**
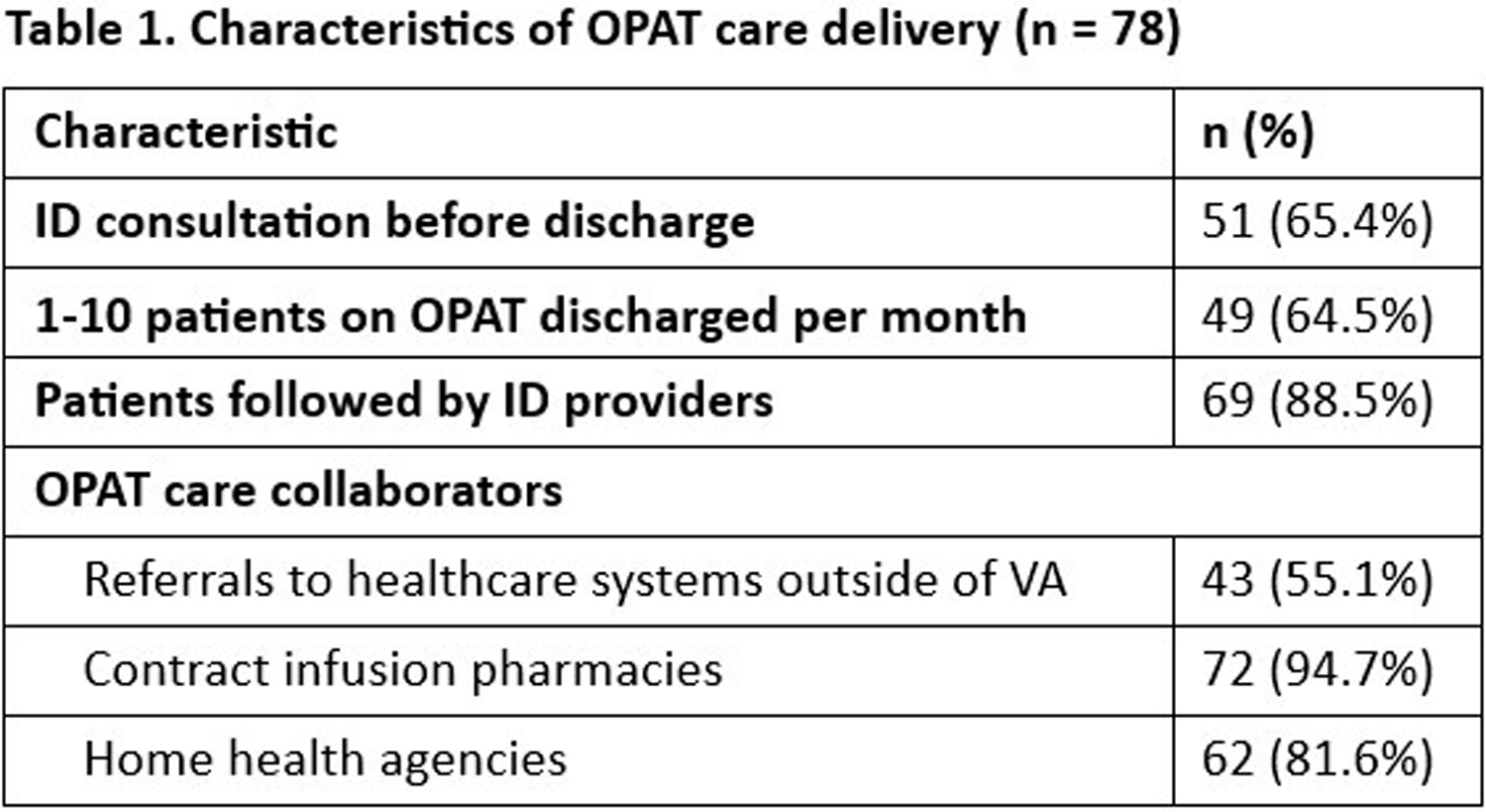

**Figure:**